# Assessment of Heavy Metals in Mexican Dietary Supplements Using Total X-Ray Fluorescence Spectrometry and Health Risk Evaluation

**DOI:** 10.3390/foods14203534

**Published:** 2025-10-17

**Authors:** Beltrán-Piña Blanca Gladiana, Santellano-Estrada Eduardo, Acosta-Montes Jorge Octavio, Cardona-Mejía Mariana, Aguilar-Maldonado Paulina, Chávez-Calderón Adriana

**Affiliations:** 1 Faculty of Nursing and Nutriology, Autonomous University of Chihuahua, Campus UACH II, Chihuahua C.P. 31125, Mexico; bbeltran@uach.mx (B.-P.B.G.);; 2 Faculty of Zootechnics and Ecology, Autonomous University of Chihuahua, Periférico Francisco, R. Almada Km. 1, Chihuahua, Chihuahua C.P. 31453, Mexico

**Keywords:** dietary supplements, heavy metals, TXRF, health-risk assessment

## Abstract

This study used Total X-ray Fluorescence Spectrometry (TXRF) to analyze toxic heavy metals, specifically lead (Pb), arsenic (As), and chromium (Cr), in 45 dietary supplement samples consumed by the Mexican population. A health risk assessment was performed using the Target Hazard Quotient (THQ), Hazard Index (HI), and Cumulative Cancer Risk (CCR). The mean concentrations of Pb, Cr, and As were found to be 1.99 ± 0.13, 26.88 ± 0.23, and 2.39 ± 0.11 mg/kg, respectively. For Pb, 80% of dietary supplements showed low EDIs compared to the reference value of 1.08 × 10^−4^ mg/Kg bw/day set by the FDA. For Cr, 100% of the analyzed products had EDIs below the limit of 0.3 mg/Kg bw/day established by EFSA. Additionally, some dietary supplements of animal origin had EDIs exceeding the reference value of 6 × 10^−5^ mg/kg bw/day set by the Integrated Risk System and EFSA for daily arsenic intake. When evaluating the variables THQ_Pb, THQ_Cr, and THQ_As using mean comparison tests to determine whether they exceeded the reference value of 1, we found that, in general, the available statistical evidence is insufficient (*p* > 0.05) to conclusively state that the dietary supplements under review surpass this reference parameter. Regarding HI values, the Animal and Vegetable category showed higher values than 1. All dietary supplements had CCR values in ranges greater than 1 × 10^−4^, indicating a probability of an individual developing cancer over their lifetime due to metal exposure. Effectively communicating these risks to consumers is crucial for promoting informed choices and improving public health.

## 1. Introduction

Similar to global trends, the Mexican population is adopting healthy habits and valuing a good quality of life, which has led to a rising demand for dietary supplements. The nutritional supplements market in the country is nearly $535,961,819 million annually, and it continues to grow [1]. Regulatory organizations in the Committee have noted issues related to the safety, efficacy, and quality of dietary supplements [2].

Dietary supplements, also known as food and nutritional supplements or nutraceuticals, are defined by the European Food Safety Authority [3]. These supplements may include one or more nutrients or pharmaceuticals, such as proteins, bioactive compounds, antioxidants, vitamins, and other ingredients [4]. Ingredients in dietary supplements can originate from both plant-based and non-vegan sources [5].

Many people think that over-the-counter dietary supplements are naturally safe, believing their benefits outweigh any possible risks [3]. However, while dietary supplements can provide many health benefits, the presence of various heavy toxic metals can significantly reduce product quality and consumer safety [2].

Obtaining raw materials, preparation, packaging, storage, and transportation are the phases involved in manufacturing food supplements. Quality control at each stage is crucial to prevent contamination, such as with heavy metals.

Heavy metals such as cadmium (Cd), lead (Pb), and arsenic (As) have been detected in dietary supplements, often exceeding recommended safety limits [6,7,8]. Other metals, such as Chromium (Cr), have also been detected as an essential nutrient required for glucose and fat metabolism; however, Cr can be toxic in large doses [9].

Heavy metal contamination in dietary supplements is inevitable due to contamination pathways throughout the supply chain [2]. Key sources include agricultural contamination from polluted water and soil, manufacturing deficiencies involving poor operational procedures and inadequately maintained equipment [6], processing-related cross-contamination, and distribution problems during transportation, storage, and packaging [10].

However, since dietary supplements are regulated as food, manufacturers are not required to present quality analyses to national or international agencies prior to sales [2]. Some nations have set strict quality control regulations such as maximum allowed limits (MAL) for metals [10]. For lead, the FDA proposes a TDI of 0.00018 mg/kg bw/day [11], while EFSA suggests 0.0005 mg/kg bw/day, reflecting different assessment approaches and safety factors. For arsenic, both EFSA and IRIS recommend a TDI of 0.00006 mg/kg bw/day [12]. Regarding chromium, the EFSA CONTAM Panel derived a TDI of 0.300 mg/kg bw/day based on the lowest No Observed Adverse Effect Level (NOAEL) from chronic oral toxicity studies in rats [13].

Regular consumption of multiple supplements can lead to cumulative exposure, raising concerns about long-term health impacts [14], especially in vulnerable groups.

### 1.1. Health Effects of Pb, As, and Cr in Vulnerable Populations

Heavy metal exposure through dietary supplements poses particular risks to vulnerable populations due to enhanced absorption, reduced detoxification capacity, and critical developmental windows. Table 1 contains the adverse effects by population and specific metal.

### 1.2. Heavy Metals in Dietary Supplements

Few studies examine heavy metals in food supplements. One study checked for Cu, Zn, Cd, Pb, and Hg in 24 dietary supplements bought in northwest Mexico. The most common toxic metals found were Pb, Cd, and Hg [2]. Another study measured manganese (Mn), copper (Cu), lead (Pb), arsenic (As), and cadmium (Cd) in 26 products containing Ginkgo biloba using graphite furnace atomic absorption spectrometry (GF-AAS). All products contained Pb, 54% had As, and 81% had Cd [33]. A different study in the Mexico metropolitan area analyzed 23 products labeled as herbal medicines, food supplements, and traditional herbal remedies. GF-AAS was used to determine metal levels. Most of the supplements contained Cu, Pb, Cd, and As [34].

There is a strong interest in conducting safety analyses on dietary supplements because these products are widely used in Mexico and globally.

Therefore, ongoing monitoring and regulation are vital to protect consumer safety. The most common techniques for analyzing heavy metals in dietary supplements include atomic emission spectroscopy with inductively coupled plasma (ICP-OES) [6], inductively coupled plasma mass spectrometry (ICP-MS) [3,7], and GFAAS [33,35].

On the other hand, a proposed protocol involved using the TXRF technique (total reflection X-ray fluorescence spectroscopy) for trace analysis of toxic metal levels in dietary supplements [9]. Its advantages include high sensitivity due to low detection limits and minimal background noise in the energy spectrum. Additionally, using an internal standard simplifies metal quantification; the sample size is small (ranging from ng to µg depending on sample preparation), and it allows for simultaneous multielement analysis [36].

The World Health Organization (WHO), the United States Environmental Protection Agency (USEPA), and the European Union (EU) have developed risk evaluation models to assess the harmful effects of both toxic and non-toxic metals from dietary supplements on human health. The variables required for risk assessment include the estimated daily intake (EDI), target hazard quotient (THQ), hazard index (HI), carcinogenic risk (CR), and cumulative carcinogenic risk (CCR) [6].

Mexico currently lacks established regulatory limits for heavy metals in dietary supplements, underscoring the urgent need for baseline data. As supplement consumption rises among at-risk populations and the potential for cumulative exposure from multiple sources grows, evaluating contamination levels becomes essential. This research provides regional data, filling a significant knowledge gap for Mexico and the Latin American region.

Therefore, the study aimed to analyze the levels of lead, arsenic, and chromium in dietary supplements found in Mexican stores using the TXRF analytical method. Concerning the intake of heavy metals through food supplements, an estimate of the potential health risk was also performed.

## 2. Materials and Methods

In this research, a validated methodology was employed to analyze Pb, Cr, and As in dietary supplement samples using TXRF, as proposed by Beltrán et al. [9].

### 2.1. Chemicals

The reagents used were of analytical grade, such as nitric acid (HNO_3_, 69% w/w, CAS No. 7697-37-2, Cat. No. JTB-9598-34), hydrochloric acid (HCl, 36.5–38% w/w, CAS No. 7647-01-0, Cat. No. JTB-9535-5), and hydrogen peroxide (H_2_O_2_, 30% w/w, CAS No. 7722-84-1, Cat. No. JTB-2186-01) (purchased from J.T. Baker in Chihuahua, Chihuahua, Mexico). Standard aqueous solutions of As (CAS No. 7440-38-2, Cat. No. AA03N-1) and Cr (CAS No. 7440-47-3, Cat. No. ICP-MS-13N-R-0.01X-1) were prepared from ICP standard stock solutions containing 1000 mg/L of each metal, which were purchased from Cresent Chemical Co., Inc. (Islandia, NY, USA). The standard solutions for Pb (1000 μg/mL, CAS No. 7439-92-1, Cat. No. ICP-29N-1) and Ga (1000 μg/mL, CAS No. 7440-55-3, Cat. No. ICP-20N-1) were obtained from Sigma Aldrich (St. Louis, MO, USA).

Calibration plots were used to determine the heavy metal content in the analyzed samples. Gallium was used as an internal standard (IS) to enhance the accuracy of the quantitative analysis.

For each element, known concentrations were prepared in 10 mL volumetric flasks and filled to the mark with 0.31 M HNO_3_ and 10 μg/L Ga (IS) solution. Ultrapure water (resistivity of 18.2 MΩ·cm) was used for all dilutions, and all glassware was cleaned routinely with 10% HNO_3_ solution.

### 2.2. Sample Collection

Dietary supplements were bought from drugstores and health food stores in Chihuahua, Mexico, during 2023–2024, selecting products identified as bestsellers by retailers. Concerning product labeling and regulatory oversight, the analyzed dietary supplements exhibited significant shortcomings in both areas: Most products studied had poor labeling practices, with limited information about ingredient details, metal levels, manufacturing data, and supplier IDs. This lack of proper labeling hindered full product traceability and informed consumer choices. None of the analyzed dietary supplements had undergone formal pre-market review by COFEPRIS (Federal Commission for the Protection against Sanitary Risks), Mexico’s health product regulatory authority.

This lack of regulatory oversight highlights the current gap in Mexican law, which only sets basic manufacturing hygiene standards without requiring heavy metal testing or content verification. These regulatory and labeling gaps emphasize the importance of independent analytical verification, as demonstrated in this study, and stress the need for comprehensive regulatory updates to safeguard consumer health. The samples were labeled for acid digestion (tablets, capsules, tea bags, and powder). For each supplement product, multiple units were pooled to ensure sample representativeness. For tablets, 20 individual tablets were crushed and thoroughly homogenized, and approximately 0.05 g of the resulting powder was taken for acid digestion. For capsules, 20 capsules were opened, their contents were mixed thoroughly, and approximately 0.05 g of the homogenized material was taken for digestion. For tea samples, one tea bag was steeped in hot water at 80 °C for 15 min. Then, 1 mL of the infusion was collected for the digestion process. A total of 45 different supplement products were analyzed in this study.

### 2.3. Sample Preparation

To remove organic materials from the food supplement samples, acid digestion was performed. First, each sample was weighed between 0.020 and 0.050 g and placed in polypropylene tubes. Next, 5 mL of H_2_O and 1 mL of HNO_3_ (69% *w*/*w*) concentrated solution were added to glass testing tubes. The reagents and sample mixtures were allowed to stand for 15 min. Then, 100 μL of H_2_O_2_ (30% *w*/*w*), 485 μL of HNO_3_ (69% *w*/*w*), and 100 μL of HCl (36.5–38% *w*/*w*) concentrated solutions were added to the tubes, which were covered with their respective stoppers. The samples were shaken with a vortex, then placed into a water bath at 80 °C for eight hours. After acid digestion, the samples were filtered to reduce undigested particles in the solutions. This step ensures that the sample contains no undigested particles, which is critical because such particles can interfere with measurements and damage the detector, as recommended by the TXRF equipment supplier. Finally, the samples were transferred into 25 mL volumetric flasks and filled to the mark with 0.31 M HNO_3_ and a 10 μg/L Ga (IS) solution.

### 2.4. TXRF Analysis

The sample introduction system uses quartz disks and a cassette to hold them. Previously, quartz discs were coated with a silicon solution in isopropanol (CAS No. 67-63-0, Cat. No. 35130.02, Serva Electrophoresis GmbH, Berlin, Germany) before the aqueous sample was deposited. An automatic micropipette was used to place a 10 µL drop of the sample on the center of the quartz disk, which was then dried at 80 °C with a hot plate to form a small sample spot at the center of the disk.

Heavy metal analyses were performed using a S2PICOFOX-TXRF spectrometer (Bruker AXS Microanalysis GmbH, Berlin, Germany), equipped with a molybdenum (Mo) x-ray tube operated at 600 μA and 50 kV. The automatic sample changer (holder) can hold up to 25 sample carriers for loading and analysis. The TXRF software, Spectra PICOFOX^®^ (Version 7, Bruker Nano GmbH, Berlin, Germany), was used for instrument control and data collection. Each sample was measured for five minutes. TXRF analyses were performed at the Autonomous University of Chihuahua, Mexico.

### 2.5. Quality Control

The analytical technique used was validated and fine-tuned by Beltran et al. [9]. However, new calibration curves, limits of detection (LOD), and limits of quantification (LOQ) were recalculated. Both the instrumental and chemical conditions, including the TXRF instrument, were precisely the same as those used by Beltrán et al. [9].

For quality control purposes: (i) blanks and samples were analyzed in triplicate during the measurement; (ii) to correct possible instrumental noise and improve the precision of quantitative analysis, gallium was used as an internal standard; (iii) calibration curves were plotted linearly (y = mx + b), with “m” denoting the calibration curve’s slope, “b” as the intercept, and “x” as the concentration (Table S1 and Figure S1, supplementary data). Linear regression equations were established, and detection limits (LOD and LOQ) were determined, with lower values indicating sufficient sensitivity (Table S2, supplementary data); (iv) five runs of analysis of the different dietary supplements were performed. In each run, two samples were selected to perform a standard addition procedure and calculate the metals’ percentage recovery.

After digestion, samples (V9, V10, A11, A12, V14, V15, V16, S27, M1, and C6) were filtered and transferred into 25 mL volumetric flasks. These samples were spiked with 625 µL of a 1000 μg/L standard solution containing Pb, Cr, and As to achieve a final concentration of 25 μg/L for each element. The flasks were then filled to the mark with 0.31 M HNO_3_ containing 10 μg/L of Ga as an internal standard (IS). The standard addition method was performed once for each of the aforementioned samples to validate the analytical process and assess matrix effects. Additionally, the same samples were analyzed without standard addition for comparison purposes and to calculate analyte recovery percentages; v) additionally, the recovery percentage was calculated in a dietary supplement (Spring Valley Chromium Picolinate), which listed a known concentration of 1000 mg/Kg of chromium per tablet.

### 2.6. Human Health Risk Assessment

The model used by Naz et al. [6] was employed to evaluate the risk of heavy metal contamination from dietary supplements on human health. For this, EDI, THQ, HI, and CCR were calculated for all metals (Pb, As, and Cr). The ATnon-cancer was set as 365 days/year times 30 years, and the adult body weight was set at 70 kg. According to USEPA recommendations, EF (exposure frequency) and ED (exposure duration) were considered as 260 days/year and 30 years, respectively [6]. The daily supplement intake was calculated based on the recommended dose listed on the supplement label [4,10].

#### 2.6.1. Estimated Daily Intake (EDI)

EDI is a widely utilized concept in chemical threat evaluation. It defines the maximum amount of a chemical an individual can ingest daily throughout their lifetime without experiencing adverse effects [4]. Going beyond this limit could result in toxic impacts. The EDI, expressed in mg.kg^−1^ bw.day^−1^, was calculated for each metal using the following Equation (1), recommended by the USEPA [6,37]:

(1) $$EDI=\frac{\left(C\times IR\times EF\times ED\right)}{\left(AT\times Bw\right)}$$

where

C = Metal concentration in the sample in mg/kg for solid samples and mg/L for liquid samples.

IR = Ingestion rate (calculated based on the recommended dose) in mg/day or mL/day.

EF = Exposure frequency (260 days per year).

ED = Exposure duration (30 years).

BW = Body weight (70 kg).

AT non-cancer = Average exposure time (EF × ED), (365 days per year × 30 years).

#### 2.6.2. Target Hazard Quotient (THQ) and Hazard Index (HI)

Based on Equation (1), the THQ was calculated for noncarcinogenic risk. This parameter was established for each metal following Equation (2), according to the USEPA:

(2) $$THQ=\frac{EDI}{TDI}$$

where

EDI = Estimated Daily Intake in mg/kg bw/day

TDI = Tolerable Daily Intake, in mg/kg bw/day, which indicates the maximum safe amount of metal intake per day through oral exposure. The TDI values for lead, arsenic, and chromium were determined in accordance with various international standards (Table 2).

The HI was calculated as the cumulative of THQ for individual metal using Equation (3); the HI value more than 1 signifies the non-carcinogenic health risk [10,39].

HI = ∑THQ (Pb, As, and Cr)(3)

#### 2.6.3. Cumulative Carcinogenic Risk (CCR)

The cancer risk (CR) of carcinogenic effects is a person’s likelihood of developing cancer during their lifetime due to exposure to metals [6]. The CR value is obtained according to Equation (4), where a CSF value for each element is needed (Table 1). At the same time, the CCR value is the sum of CRs of individual metals according to Equation (5).

CR_Metal_ = EDI_Metal_ × CSF_Metal_ (4)

CCR = ∑CR (Pb, As, and Cr)(5)

where

CSF = Cancer Slope Factor in (mg/kg bw/day)^−1^

EDI = Estimated Daily Intake in mg/kg bw/day

### 2.7. Statistical Analysis

Measuring the concentrations of heavy metals in dietary supplements is crucial for protecting consumer health and avoiding potential health risks. In Mexico, only a few studies have evaluated the health risks associated with consuming dietary supplements [2,33,34]. To achieve this goal, a chemometric modeling approach was used, incorporating various analytical techniques, including Pearson Correlation Coefficient (PCC), T-test, Principal Component Analysis (PCA), and Hierarchical Cluster Analysis (HCA), Univariate (ANOVA), and Multivariate Analysis of Variance (MANOVA). The variables selected for this study included the estimated daily intake of Pb, Cr, and As, labeled as EDI_Pb, EDI_Cr, and EDI_As, respectively. Additionally, the Hazard Index (HI) was used in the analysis. Statistical analyses were performed using Stata Statistical Software version 18.0 (StataCorp LLC, College Station, TX, USA).

## 3. Results

### 3.1. Dietary Samples Characteristics

Forty-five dietary supplements were purchased from local supermarkets and health food stores in Chihuahua, Mexico. The selection of these dietary supplements was conducted using systematic sampling from licensed distributors, prioritizing products identified as bestsellers to ensure public health relevance. Five different supplements were systematically selected from each retail location across five major categories (minerals, vegetable, animal, synthetic, and combination supplements). The sample size of 45 supplements provides adequate statistical power for detecting meaningful contamination differences between supplement categories, consistent with established protocols in international supplement contamination research [6,7]. While this study represents a regional assessment focused on northeastern Mexico rather than a comprehensive national market analysis, the findings provide essential baseline data for an understudied geographic region.

The samples were coded according to their origin: Vegetable was coded as V (25 samples), Minerals as M (3 samples), Synthetic as S (6 samples), Combined as C (3 samples), and Animal as A (8 samples). Table 3 represents the sample ID, serving weight, daily recommended dosage as indicated on each label, ingestion rate (IR), and therapeutic indication.

### 3.2. Quality Control

The stability of the internal standard measurements was mandatory in this study since this ensured the absence of instrumental noise and improved the data quality. The reproducibility of gallium measurements expressed as relative standard deviation (RSD) was 5% demonstrating a satisfactory value following FDA guidelines (% RSD < 7) [9]. This value was obtained from five consecutive measurements of a standard of 10 µg/L of gallium per day for five days (*n* = 25).

Method validation parameters demonstrated acceptable analytical performance: detection limits (LOD) of 0.05, 0.03, and 0.08 µg/L for Pb, As, and Cr, respectively, with corresponding quantification limits (LOQ) of 0.15, 0.10, and 0.25 µg/L. Linearity was confirmed across the concentration range of 0.1–100 µg/L for all analytes (Table S1, Figure S1, Supplementary data).

Accuracy was evaluated through recovery studies using dietary supplement reference materials, yielding recoveries between 83–107% for all elements. All validation parameters comply with international guidelines (ICH Q2(R1)) for analytical method validation, ensuring reliable quantitative results for heavy metal determination in dietary supplements (Table 4).

Recovery values exceeding 100% were observed for some elements and can be attributed to several analytical factors. Matrix enhancement effects, where sample components increase analyte signal intensity, may contribute to apparent over-recovery. Additionally, potential cross-contamination during sample preparation and memory effects between consecutive analyses were considered as possible sources. Specifically, four of the samples showed recoveries > 100% for Cr and As, with maximum recovery values between 101–107%. Nevertheless, all recovery values remained within the acceptable range of 70–150% established by USP 233 for spike recovery studies, confirming the analytical method’s reliability despite these minor deviations [40]. Furthermore, a percentage recovery of 107% was obtained for the Chromium Picolinate sample, which was used as the reference material.

### 3.3. Application to Real Samples

The US Pharmacopeia, in chapter 2232, includes limits of individual contaminants based on a maximum daily intake of 10 g of a dietary supplement. This study used the USP values of 1.5 mg/Kg and 1 mg/Kg for As and Pb, respectively, to compare with the concentrations found in the dietary supplements. Regarding Cr, in Chapter 232, the USP set a concentration limit of 1100 mg/Kg for components used in oral drug products; this value was selected as a reference in this study [41].

The contents of metals in dietary supplements are summarized in Table 5. The mean levels for Pb, Cr, and As were 1.99 ± 0.13, 26.88 ± 0.23, and 2.39 ± 0.11 mg/Kg, respectively. Cr is the most abundant element among all the studied metals.

The T-test revealed statistically significant superiority of lead concentrations observed in some of the food supplements with respect to the established maximum permissible limit (*p* < 0.05).

Regarding the average concentration of arsenic in food supplements, it was concluded that there is not enough evidence to affirm that dietary supplements exceed the maximum limit established for As (*p* > 0.05).

In comparing the mean chromium concentration to the maximum concentration, there is insufficient evidence to claim that the supplement exceeds the established maximum limit for Cr (*p* > 0.05).

#### 3.3.1. Lead

The highest levels in mg/Kg of Pb above its reference value (1 mg/Kg) [42] were 9.03, 8.17, 7.20, 6.32, and 4.51 for samples A11, V18, A45, A47, and M1, respectively (Table S3, Supplementary data). While the range concentration in mg/Kg for each category was as follows: 0.39–9.03, 0.07–1.75, 1.08–4.51, 0.28–1.81, and <LOD–8.17 for animal, combined, mineral, synthetic, and vegetal, respectively. Other research reported Pb concentrations in dietary supplements of 1.70, 0.84, and 3.7 mg/Kg [3,6,43]; these values were comparable to some of the Pb levels in this study. In Mexico, Pb concentrations were reported to be similar (4.81 mg/Kg) to those found in various dietary supplements (M1 and V9) [2].

The supplement with the highest Pb content was Sample A11 (9.03 mg/Kg) (Table S3, Supplementary data), popularly known in Mexico as “Víbora de cascabel” (Rattlesnake), which is offered to the consumer as a blood purifier, for acne, allergies, anemia, asthma, arthritis, gallstones, kidney stones, cancer, diabetes, among others. It is a powder obtained by grinding the skin of the rattlesnake (*Crotalus* spp.) after having gone through a drying process. Notably, despite the multiple health benefits offered by the product, the Pb content specifically in this sample was nine times higher than that established by the USP (Chapter 2232). The health problem lies in the fact that chronic Pb exposure in adults can cause multiple neurological disorders, such as nerve disorders, memory or concentration problems, and lack of muscular coordination, among others [14]. On the other hand, lead can compete with calcium absorption, affecting bone mineralization. Additionally, the formation of reactive oxygen species (ROS) for Pb intoxication leads to cellular damage [44].

#### 3.3.2. Arsenic

Arsenic toxicity impacts the cardiovascular, dermatologic, nervous, hepatobiliary, renal, gastrointestinal, and respiratory systems [45]. The harmfulness of As depends on the level of exposure, frequency, duration, and both organic and inorganic forms, as well as age, gender, and genetic and nutritional factors [44].

In this study, the average arsenic (As) concentrations (mg/Kg) in the analyzed samples were 1.57, 0.67, 3.63, 0.60, and 0.04 for the following categories: Animal, Mineral, Vegetal, Combined, and Synthetic, respectively (Table 5). The Animal category exceeded its reference value (1.57 versus 1.5 mg/Kg), while the Vegetal category was twice the reference value set by the US Pharmacopeia [42]. In contrast, arsenic concentrations in dietary supplements from Lebanon, the USA, and Poland were 0.26, 0.37, and 0.26 mg/Kg, respectively, which are lower than the results found in the current study [10,46,47].

Sample V13, a seaweed supplement, presented the highest arsenic levels (39.93 mg/Kg) (Table S3, Supplementary data), which fell within the concentration range of 2.3–141 mg/Kg reported in several studies [48,49]. Some supplements use seaweed as a raw material, especially for weight loss. However, seaweed is a food source obtained mainly from the coasts, where both environmental and industrial pollution allow heavy metals such as arsenic to bioaccumulate in plants and fish [49].

#### 3.3.3. Chromium

The Cr content in commercial dietary supplements could be found in concentrations of 250, 500, 1000, and 1100 mg/Kg. However, the type of diet, the chemical form of the molecule, and the pharmaceutical form of preparations significantly influence the bioavailability of Cr. The determined relative bioavailability ranges from 2.97 to 3.70% [50]. The Cr(III) is considered an essential nutrient and may help maintain standard glucose tolerance. Nevertheless, there are safety concerns regarding its supplemental intake for a large period due to increasing evidence of genotoxicity [6].

Regarding chromium, no established maximum permissible limits exist for dietary supplements in major regulatory frameworks, due to its dual nature as both an essential micronutrient and a potential toxicant. However, a reference Cr value (1100 mg/Kg) was taken from the USP in Chapter 232, for components used in oral drug products, to compare with our results [41]. Analytical results from the analyzed samples were substantially lower than the USP standard (*p* > 0.05).

The mean results of the chromium analysis for the Combined, Animal, and Synthetic categories were 7.69 mg/Kg, 14.75 mg/Kg, and 1.12 mg/Kg, respectively (Table 5). These values were lower than those reported by Mihai et al. in vitamin supplements, which ranged between 9.17 and 19 mg/Kg [51]. On the other hand, high Cr concentrations were found for the Mineral (49.86 mg/Kg) and Vegetable (36.49 mg/Kg) categories.

Samples M1, V16, V19, and V44 showed the highest concentrations of chromium: 131.46, 135.41, 150.01, and 127.97 mg/Kg, respectively (Table S3, Supplementary data). These samples share the characteristic that they are indicated for weight loss. Weight loss supplements are very popular; women (44.9%) are the primary consumers [52].

Although the Cr concentrations were within a safe range, the issue arose because Cr (III) has low membrane permeability and cannot penetrate the cell membrane. As a result, it remains inside the cell where it can bind to DNA, leading to genetic damage and genomic instability. Another stable oxidation state of chromium is Cr (VI), which can enter many types of cells and, under physiological conditions, can be reduced by hydrogen peroxide (H_2_O_2_), glutathione (GSH) reductase, ascorbic acid, and GSH to form reactive intermediates such as Cr (V), Cr (VI), thiol radicals, and hydroxyl. These species could attack DNA, proteins, and membrane lipids, disrupting cellular integrity and functions [53]. Exposure to chromium has been linked to the development of various cancers, including those of the lungs, larynx, bladder, bone, stomach, kidneys, testicles, and thyroid [14].

### 3.4. Regulatory Oversight of Dietary Supplement Production and Distribution in Mexico

A recent rise in dietary supplement consumption has been seen in Mexico. According to data, revenues from the vitamin and mineral market are expected to reach around US$344.99 million by 2025 [54].

Mexican law regulates dietary supplements through the General Health Law, specifically in Article 215, which defines them as products made from herbs, plant extracts, or nutritional concentrates intended to supplement a person’s regular diet [55].

The Federal Commission for the Protection against Sanitary Risks (COFEPRIS) serves as the primary supervisory agency, enforcing various operational requirements. Manufacturers must adhere to specific regulations such as NOM-051-SCFI/SSA1-2010 and NOM-086-SSA1-1994. Labeling is mandatory and must be in Spanish, including detailed nutritional information and warnings about responsible use [56].

For marketing purposes, companies must submit a “Notice of Operation” 30 days in advance. Products must display the statement “This product is not a medicine.” An Advertising Permit is also required and takes about two months to process.

It must be confirmed that the ingredients are not on the list of restricted substances (ephedrine, procaine, and hormones). Mexico’s 2009 dietary supplement regulations only specify manufacturing hygiene standards without setting maximum limits for heavy metals [56]. In the case of chromium, an intake of 200 g per day is allowed.

The levels of heavy metals in the analyzed dietary supplements were within the maximum permissible limits set by major international regulatory agencies (FDA, EFSA, and IRIS). However, the statistical analysis revealed that, in general, Pb concentrations significantly exceeded the reference standards of these organizations (*p* < 0.05), highlighting a potential health risk to consumers that requires regulatory action.

The situation is worsened by long-term consumption patterns without medical supervision, based on the misconception that these products are entirely safe [43]. Furthermore, the lack of immediate symptoms and the slow development of toxic effects make early detection of metal-related health issues especially difficult [3].

### 3.5. Human Health Risk Assessment

#### 3.5.1. Estimated Daily Intake

Table 6 displays the EDI of Pb, Cr, and As in dietary supplements, evaluating potential lifetime health risks to humans. This study used the manufacturer’s recommended daily dose.

The worldwide analysis of estimated daily intakes revealed the following:

For Pb, 80% of dietary supplements showed low EDIs compared to the reference value of 1.08 × 10^−4^ set by the FDA [11], with the exception of those of Animal origin (*p* = 0.005). The *T*-test analysis indicated that the mean EDI_Pb by supplement category did not significantly exceed the FDA’s daily intake limit (*p* > 0.05), suggesting compliance with current regulatory standards for lead exposure through dietary supplements. One study conducted in Mexico reported similar findings in the analysis of dietary supplements [34]. However, our results showed low EDIs compared to the highest EDI of 5.46 × 10^−3^ observed in research from Romania [35].

For Cr, 100% of the analyzed products permitted EDIs less than 0.3 mg/Kg bw/day established by the EFSA [13]. The T-test concluded that there is insufficient evidence to confirm that the dietary supplements analyzed exceed the maximum limit established for daily chromium intake (*p* > 0.05). However, to mention a few examples, in this study, the samples V20, M1, V16, and V32 had EDIs ranging from 3.39 × 10^−3^ to 2 × 10^−2^ mg/kg bw/day (Table S4, Supplementary data). These values were higher than those reported by Naz et al. (7.08 × 10^−7^ to 5.28 × 10^−5^) in a similar study [6].

EDI values for the toxic metal As (inorganic) ranged from 1.07 × 10^−6^ mg/kg bw/day for the lowest value, a pharmaceutical herbal product (V33), to 1.7 × 10^−3^ mg/kg bw/day for the highest, a traditional herbal remedy (V13) (Table S4, Supplementary data).

Additionally, some dietary supplements of animal origin had EDIs exceeding the reference value of 6 × 10^−5^ mg/kg bw/day set by the Integrated Risk System and EFSA for daily arsenic intake [12,38]. Similarly, other studies from Mexico reported the highest EDI value for arsenic as 1.98 × 10^−2^ for traditional herbal remedies used as dietary supplements [34]. The level of exposure to toxic metals can vary depending on the recommended dosage by the manufacturer and the average body weight [6]. Nonetheless, real-world consumption may vary considerably, with consumers possibly exceeding these doses, using multiple supplements at once, or following irregular intake patterns. To achieve more accurate exposure assessments, future research should include consumer survey results or market consumption data, aligning with EFSA guidance on dietary exposure analysis [57].

#### 3.5.2. Total Hazard Quotient and Hazard Index

The target hazard quotient (THQ) indicates the potential risks to human health of the contaminants and was calculated for Pb, Cr, and As in dietary supplements (Table S4, Supplementary data). Consumers may not experience negative health consequences if the THQ value exceeds 1. On the other hand, a THQ of one or higher indicates possible consumer risk exposure [58].

The THQ for Pb and Cr were less than 1 (THQ < 1) for all dietary supplements analyzed (*p* > 0.05). In the arsenic analysis, THQs of 18.88 and 20.32 were obtained for V41 and V13 samples, respectively (Table S4, Supplementary data), suggesting that the ingestion of these products may pose a risk to human health, due to their high As content. The As contamination is more common in dietary supplements of vegetal origin, as the primary routes of exposure to the metal are food, water, soil, and air [34], indicating that greater scrutiny is required for these products on sale in Mexico.

**Figure 1:**
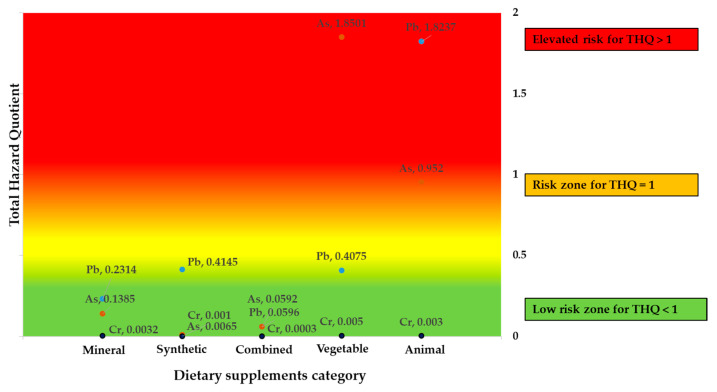
Toxicological Risk Heat Map: THQ mean values in each dietary supplement category: Mineral (*n* = 3), Synthetic (*n* = 6), Combined (*n* = 3), Vegetable (*n* = 25), Animal (*n* = 8).

When evaluating the variables THQ_Pb, THQ_Cr, and THQ_As using means comparison tests to determine whether they exceeded the reference value of 1, we found that, in general, the available statistical evidence is insufficient (*p* > 0.05) to categorically state that the food supplements under study exceed this reference parameter. However, Figure 1 shows the dietary supplements in a heat map, where red indicates nutritional supplements with high THQ values.

HI’s results are presented in Table S4, Supplementary data. The Animal and Vegetable category showed higher values than 1 (Table 7). The T-test indicated there was insufficient evidence to confirm that the HI for dietary supplements exceeds the unit (*p* < 0.05), except for the Animal category (*p* ˂ 0.05).

Similar results were reported by Bandara et al. and Naz et al., where HI values were less than 1 [6,47]. Conversely, in another study conducted in Mexico, the HI values were higher at 3.75, indicating a comparatively elevated level of potential health risk [34]. In fact, in this research, it had HI > 1, such as A11 (5.84), V15 (7.89), V41 (18.88), and V13 (20.74), to mention a few of them (Table S4, Supplementary data).

The HI considers and estimates that the ingestion of a particular type of food would result in simultaneous exposure to several toxic elements. Although individual THQs calculated for individual components in the dietary supplements were below 1, the cumulative effect suggests adverse health effects [59].

#### 3.5.3. The Cumulative Cancer Risk (CCR)

The cumulative cancer risk associated with dietary supplements contaminated with heavy metals is a growing concern.

**Figure 2:**
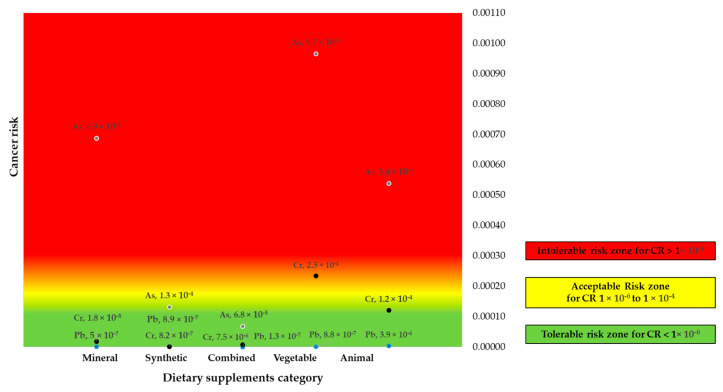
Cancer risk Heat Map: CR values. Values are expressed as the mean of CR values for each category of supplements. Mineral (*n* = 3), Synthetic (*n* = 6), Combined (*n* = 3), Vegetable (*n* = 25), Animal (*n* = 8).

This research calculated the cancer risk (CR) presented to human health by individual potential carcinogenic metals. Figure 2 shows a heat map with the CR values for each dietary supplement category. Values of CR less than 1 × 10^−6^ are considered tolerable (green area), while those falling between >1 × 10^−6^ and 1 × 10^−4^ are considered within an acceptable range (yellow area). Finally, values exceeding 1 × 10^−4^ were considered intolerable (red area) [6,37].

This research indicates that while many dietary supplements may contain heavy metals like Pb, Cr, and As, the health risks from typical consumption levels are often below critical thresholds. However, cumulative exposure from multiple supplements can pose significant risks. It is important to note that, in general, there was a tendency to exceed the level (CCR > 1 × 10^−4^) (*p* = 0.0955), which was marked in those of plant origin (*p* = 0.097).

Then, the CCR, which may promote carcinogenic effects depending on the exposure dose, was calculated based on the ingestion of Pb, Cr, and As. Results were presented in Table S3, Supplementary data.

The CCRs obtained in this study were 7.1 × 10^−4^, 1.3 × 10^−4^, 7.6 × 10^−5^, 1.2 × 10^−3^, and 6.6 × 10^−4^ for Mineral, Synthetic, Combined, Vegetable, and Animal categories, respectively. All dietary supplements had CCR values in ranges greater than 1 × 10^−4^, with a probability of an individual developing cancer over their lifetime as a result of exposure to metals. It was ?not possible to compare these results with other studies conducted in Mexico because they did not evaluate a CCR value. However, similar to our results, most of the analyzed dietary supplements contained values in the ranges of intolerable levels [35]. In contrast, other investigations found that the CCR of every sample fell within an acceptable range [6].

### 3.6. Multivariate Analysis

A PCC analysis was performed (Table S5, Supplementary data) to determine the degree of linear association between response variables, including EDI_Pb, EDI_Cr, EDI_As, and HI. Pearson’s correlation analysis (*n* = 45) showed a powerful and highly significant positive correlation between daily As intake and the hazard index (r = 0.97, *p* < 0.0001), indicating that arsenic is the primary contributor to HI from the consumption of these dietary supplements.

According to the PCA, the table of total explained variance obtained from the principal component analysis (Table S6, Supplementary data) indicates that two components, which explain 76.23% of the global variability, were retained.

By its coefficients, Principal Component 1 explains 50.53% of the total variation in the four original variables by itself. The most substantial contribution to Principal Component 1 is made by the variables HI and EDI_As. This component is shown as an index that contrasts (separates) supplements with high levels in EDI_As, HI, EDI_Pb, and low values in EDI_Cr (example V13, V41, V15, A11, A12, and A45) against supplements with high values in EDI_Cr and low values in HI and EDI_As (example V32, V19, and V39).

The strongest contribution to Principal Component 2 is made by the variables EDI_Cr and EDI_As, and this component contrasts (separates) supplements with high levels of EDI_Cr, EDI_Pb, and low values of EDI_As (example V32, V19, A45, V15, and A47), versus supplements with high values of EDI_As and low values of EDI_Cr and EDI_Pb (example V41 and V13).

**Figure 3:**
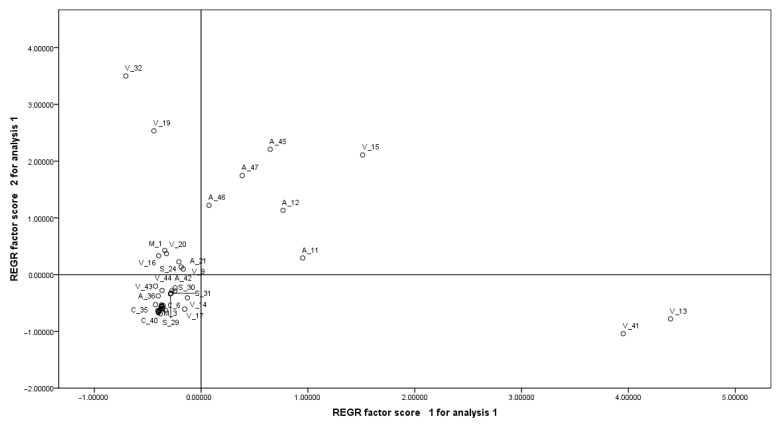
Demonstration of PCA; *Y axis*, Principal component 2, and *X axis*, Principal component 1.

Figure 3 was obtained when plotting the first two principal components.

The high variability recorded in the plant-based supplements (as can be seen in their coefficient of variation in Table 6) generates an extremely heterogeneous behavior of these supplements in multidimensional terms. This is why these supplements are located both at the center and at the extremes of Figure 3. This contrasts with the animal-based supplements, which show a smaller multivariate distance among themselves and are mainly positioned at the center of this figure.

**Figure 4:**
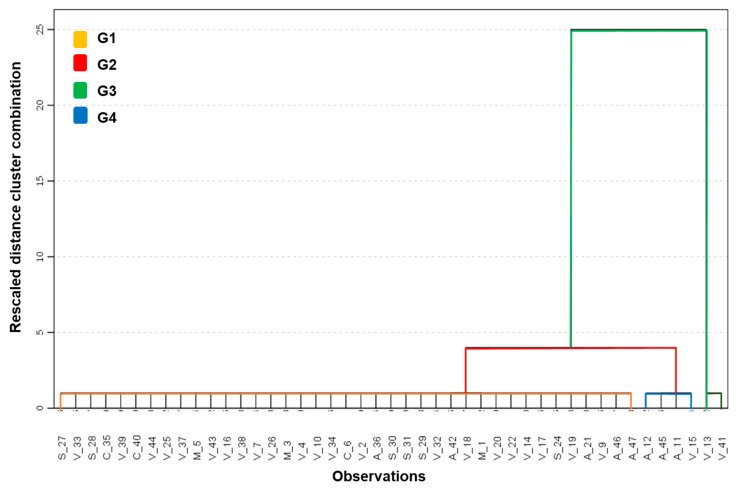
Dendogram from Cluster Analysis. Colors represent different groups (G1, G2, G3, G4) formed by hierarchical clustering.

A cluster analysis was conducted to evaluate the feasibility of grouping the supplements into clusters that are internally homogeneous and significantly different from each other (Figure 4). The R^2^ criteria and the cubic clustering criterion (CCC) were used. Four groups (Table S7, Supplementary data) were formed, resulting in an R^2^ of 0.95. These groups were found to differ in a multivariate manner using Wilks’ Lambda statistic from MANOVA (*p* < 0.0001) and also in a univariate manner, with ANOVAs indicating highly significant differences (*p* < 0.0001) for the four variables examined.

Group 1 includes dietary supplements that increase lead and chromium intake, while groups 2 and 3 include those that increase chromium intake (Figure 4).

Group 4 consists of dietary supplements that contribute to higher arsenic intake. Regarding the hazard index, group 4 consisted of dietary supplements that contribute to a very high hazard index (HI = 19.81) (confirmed by the correlation r = 0.97, *p* = 0.0001 with HI).

Due to the high variability recorded in the plant-based supplements for the variables considered, some of these supplements were located across the four generated groups, highlighting supplements v13 and v14, which, given their extremely high values in As and HI, formed a single group; conversely, the animal-based supplements, with a more homogeneous multivariate behavior, were mainly located in two of the groups.

### 3.7. Novel Contributions of This Study

This study provides three distinct contributions to the scientific understanding of heavy metal contamination in dietary supplements:

- Regional Knowledge Gap: This study provides the first comprehensive analysis of lead, arsenic, and chromium in dietary supplements from northeastern Mexico, filling a crucial geographic data gap in North American contamination patterns.
- Micronutrient Toxicity Focus: Novel measurement of chromium as both a needed nutrient and potential toxin, providing baseline data for a metal rarely tracked in supplement quality control despite its dual role.
- Methodological Innovation: First application of Total Reflection X-ray Fluorescence (TXRF) for simultaneous multi-element detection in Mexican dietary supplements, demonstrating comparable performance to ICP-MS with significant environmental and economic advantages.
- Regulatory Impact: Direct evidence supporting the urgent need to update Mexico’s 2009 regulatory framework, which currently lacks heavy metal limits, providing a scientific foundation for evidence-based policy development.

These findings directly inform public health policy, clinical nutrition practice, and analytical chemistry applications, representing a significant advancement in supplement safety research for Latin America.

## 4. Conclusions

This study successfully analyzed lead, arsenic, and chromium in 45 dietary supplements from Mexico using TXRF methodology, providing the first comprehensive regional assessment of heavy metal contamination in this understudied market. Risk assessments were conducted for all elements with guidance values obtained from reports published by the European Food Safety Authority (EFSA), the FDA, and the Integrated Risk Information System (IRIS). The significance of the study extends beyond detecting contamination to address critical regulatory gaps in Mexico’s dietary supplement oversight. Our findings offer essential baseline data for COFEPRIS to establish science-based maximum permissible limits, which are currently missing from Mexican regulations. One limitation of the study includes geographic restriction to northeastern Mexico and limited sample representation of the broader market. Although most of the analyzed dietary supplements did not exceed the tolerable daily intake, the researchers recommend implementing consumer education programs with population-specific dosage guidelines, mandatory source disclosure, and accessible analytical certificates. Future research should broaden geographic sampling throughout Mexico, perform speciation analysis to differentiate between various chemical forms of these elements, and enhance exposure modeling for vulnerable populations by including age-specific consumption habits and physiological factors.

## Figures and Tables

**Table 1:** Effects on different populations and specific metals.

Vulnerable Group	Metal Exposure		
	Lead	Arsenic	Chromium
Pregnant Women	Fetal neurodevelopment damage, preterm birth, and low birth weight [15].	Gestational diabetes, miscarriage risk, and DNA damage [16].	Low birth weight risk (female infants), metabolic effects [17,18].
Lactation mothers	Transfer via breast milk, infant neurotoxicity [19,20].	Colostrum accumulation, immune system disruption [19].	Transfer via breast milk, developmental effects [19,21].
Children	Cognitive impairment, developmental delays, and academic performance [22].	Attention/memory deficits, reasoning impairment [23].	Neuropsychological development impairment [18,24].
Adolescent population	Altered stress responses, cardiovascular implications [25,26].	Reduced IQ, processing speed deficits, and language impairment [23,27].	Genotoxicity, oxidative stress, DNA damage [28].
Elderly people	Cognitive decline, hypertension, renal dysfunction [29,30].	Cardiovascular aging, cognitive deterioration, and accelerated aging [31,32].	Oxidative stress and biochemical alterations [18].

Effects based on chronic exposure studies.

**Table 2:** TDIs and CSFs values for each metal analyzed.

Metal	TDIs (mg/kg bw/day)	Slope Factor, CSF (mg/kg bw/day)^−1^	Reference
Pb	^a^ 1.8 × 10^−4^	0.0085	[6,11]
As	^b^ 6 × 10^−5^	1.5	[12,38]
Cr	^c^ 0.3	0.5	[13,38]

^a^ Interim Reference Levels (IRLs) set by the FDA for Pb. ^b^ TDI set by the Integrated Risk System and EFSA for As. ^c^ TDI set by the EFSA for Cr.

**Table 3:** Characteristics of dietary supplements.

Sample ID	* Dosage per Day	Serving Weight (Kg)	IR (Kg/Day)	Therapeutic Indication
A11	6	0.0004	0.002	Blood detoxifier
A12	1	0.03	0.03	Muscle development
A21	2	0.015	0.030	Improve the immune system
A36	6	0.0004	0.002	blood detoxifier
A42	1	0.03	0.030	Improve muscle development
A45	3	0.003	0.009	Muscle activity and cell growth
A46	3	0.003	0.009	Muscle activity and cell growth
A47	3	0.003	0.009	Muscle activity and cell growth
C35	1	0.001	0.001	Weight loss
C40	1	0.0005	0.0005	Booster energy
C6	2	0.0008	0.002	Weight loss
M1	2	0.001	0.002	Booster energy
M3	3	0.0005	0.002	Weight loss
M5	2	0.0005	0.001	Booster energy
S24	1	0.040	0.040	Relaxing of the blood vessels
S27	2	0.0005	0.001	Regulation of the sleep cycle
S28	1	0.0006	0.0006	Booster energy
S29	1	0.005	0.005	Increased muscle mass
S30	1	0.005	0.005	Increased muscle mass
S31	1	0.005	0.005	Increased muscle mass
V10	3	0.0005	0.002	Improve glucose level
V13	6	0.0005	0.003	Improve the immune system
V14	6	0.0005	0.003	Weight loss
V15	1	0.03	0.033	Improve the immune system
V16	4	0.0005	0.002	Weight loss
V17	2	0.0005	0.001	Weight loss
V18	2	0.0005	0.001	Antioxidant
V19	6	0.001	0.006	Weight loss
V2	1	0.001	0.001	Weight loss
V20	3	0.001	0.004	Improve digestion
V22	1	0.025	0.025	Improve the immune system
V25	1	0.002	0.002	Improve digestion
V26	2	0.0005	0.001	Antioxidant
V32	6	0.001	0.006	Weight loss
V33	3	0.0005	0.002	Improve glucose level
V34	2	0.0005	0.001	Antioxidant
V37	3	0.001	0.003	Weight loss
V38	6	0.0005	0.003	Weight loss
V39	3	0.001	0.004	Improve digestion
V4	1	0.002	0.002	Improve digestion
V41	6	0.0005	0.003	Improve the immune system
V43	3	0.001	0.003	Improve digestion
V44	1	0.001	0.001	Weight loss
V7	3	0.001	0.004	Improve digestion
V9	3	0.001	0.003	Improve digestion

* Dietary supplements were presented in tablets, capsules, powder, and liquid form.

**Table 4:** Results of the recovery study on dietary supplements.

^a^ Sample ID	Standard Addition (µg/L)	^b^ Metal Concentration (µg/L)			% Recovery		
		Pb	Cr	As	Pb	Cr	As
V9	0	4.03 ± 0.17	28.94 ± 0.17	0.40 ± 0.09	86	104	96
	25	25.52 ± 1.28	55.50 ± 0.40	25.02 ± 0.52			
V10	0	2.16 ± 0.07	0.397 ± 0.001	<LOD	95	96	91
	25	25.94 ± 0.56	24.51 ± 0.15	22.65 ± 0.37			
A11	0	3.83 ± 0.23	73.07 ± 0.29	0.70 ± 0.13	86	96	98
	25	25.29 ± 0.22	97.13 ± 0.44	25.21 ± 0.34			
A12	0	1.77 ± 0.03	0.3894 ± 0.0002	0.47 ±0.04	92	95	103
	25	24.77 ± 0.32	24.10 ± 0.13	26.23 ± 0.36			
V14	0	3.82 ± 0.12	0.414 ± 0.001	2.26 ± 0.06	94	102	97
	25	27.29 ± 0.84	26.04 ± 0.24	26.49 ± 0.64			
V15	0	2.43 ± 0.09	0.72 ± 0.01	<LOD	97	101	104
	25	26.57 ± 0.87	25.95 ± 0.14	26.03 ± 0.18			
V16	0	3.34 ± 0.02	151.66 ± 0.09	0.08 ± 0.02	96	96	99
	25	27.29 ± 0.24	175.61 ± 0.79	24.76 ± 0.61			
S27	0	0.26 ± 0.02	1.85 ± 0.04	0.21 ± 0.04	96	89	100
	25	24.26 ± 0.96	24.09 ± 0.40	25.29 ± 0.11			
M1	0	5.60 ± 0.70	163.01 ± 0.39	0.26 ± 0.02	83	107	106
	25	26.40 ± 0.20	189.70± 0.80	26.75 ± 0.36			
C6	0	2.65 ± 0.07	12.88 ± 0.42	0.09 ± 0.02	94	93	104
	25	26.15 ± 0.18	36.20 ± 0.20	26.10 ± 0.30			

^a^ The results are reported as the Pb, Cr, and As concentrations in the analyzed solutions. ^b^ Results are expressed as the mean value ± s (*n* = 3). s, standard deviation.

**Table 5:** Heavy Metal Concentrations by dietary supplement category.

Category	*n*	Heavy Metal Concentration (mg/Kg)		
		Pb Mean ± SD (Range)	Cr Mean ± SD (Range)	As Mean ± SD (Range)
Vegetable	25	1.72 ± 1.72 (<LOD–8.17)	36.49 ± 60.18 (<LOD–233.20)	3.63 ± 10.58 (<LOD–39.93)
Mineral	3	2.41 ± 1.84 (1.08–4.51)	49.9 ± 71.0 (2.15–131.46)	0.67 ± 0.59 (0.21–1.34)
Animal	8	3.70 ± 3.46 (0.39–9.03)	14.75 ± 15.45 (<LOD–39.56)	1.57 ± 3.98 (<LOD–11.41)
Synthetic	6	1.25 ± 0.71 (0.29–1.81)	1.12 ± 0.89 (0.42–2.44)	0.04 ± 0.09 (<LOD–0.23)
Combined	3	0.69 ± 0.92 (0.07–1.75)	7.69 ± 7.25 (0.08–14.52)	0.60 ± 0.83 (0.06–1.55)
Total	45	1.99 ± 0.13 (0.07–9.03)	26.88 ± 0.23 (0.08–233.20)	2.39 ± 0.11 (0.03–39.93)

**Table 6:** Estimated Daily Intake (EDI) for lead, chromium, and arsenic.

Variable	Origin	Mean	Standard Deviation	Min	Max	Variability Coefficient (%)
EDI_Pb	Animal	4.61 × 10^−4^	3.26 × 10^−4^	1.4 × 10^−5^	9.25 × 10^−4^	70.68
	Combined	1.50 × 10^−5^	2.30 × 10^−5^	1 × 10^−6^	4.10 × 10^−5^	150.95
	Mineral	5.80 × 10^−5^	6.10 × 10^−5^	2.3 × 10^−5^	1.29 × 10^−4^	104.23
	Synthetic	1.05 × 10^−4^	8.40 × 10^−5^	4 × 10^−6^	2.30 × 10^−4^	80.60
	Vegetable	1.03 × 10^−4^	2.09 × 10^−4^	NA	1.06 × 10^−3^	202.73
EDI_Cr	Animal	1.08 × 10^−3^	8.33 × 10^−4^	NA	2.34 × 10^−3^	77.30
	Combined	1.36 × 10^−4^	1.17 × 10^−4^	1 × 10^−6^	2.07 × 10^−4^	86.29
	Mineral	1.38 × 10^−3^	2.07 × 10^−3^	3 × 10^−5^	3.76 × 10^−3^	150.17
	Synthetic	2.64 × 10^−4^	5.68 × 10^−4^	4 × 10^−6^	1.42 × 10^−3^	215.35
	Vegetable	1.93 × 10^−3^	4.60 × 10^−3^	NA	2 × 10^−2^	237.99
EDI_As	Animal	8 × 10^−5^	1.41 × 10^−4^	NA	3.91 × 10^−4^	175.28
	Combined	5 × 10^−5^	5 × 10^−6^	1 × 10^−6^	1.10 × 10^−5^	106.36
	Mineral	1.20 × 10^−5^	7 × 10^−6^	6 × 10^−6^	1.90 × 10^−5^	57.91
	Synthetic	1 × 10^−6^	1 × 10^−6^	NA	3 × 10^−6^	244.95
	Vegetable	1.56 × 10^−4^	4.55 × 10^−4^	NA	1.71 × 10^−3^	291.74

NA. Not applicable.

**Table 7:** Hazard Index for dietary supplement origin.

Variable	Origin	Mean	Standard Deviation	Min	Max	Variability Coefficient (%)
HI	Animal	2.78	2.12	1.61 × 10^−1^	5.87	76.38
	Combined	1.19 × 10^−1^	6.86 × 10^−2^	4.46 × 10^−2^	1.80 × 10^−1^	57.57
	Mineral	3.73 × 10^−1^	1.96 × 10^−1^	2.09 × 10^−1^	5.90 × 10^−1^	52.47
	Synthetic	4.22 × 10^−1^	3.26 × 10^−1^	5.20 × 10^−2^	9.12 × 10^−1^	77.34
	Vegetable	2.26	5.51	NA	2.08 × 10^1^	243.44

NA. Not applicable.

## Data Availability

The original contributions presented in this study are included in the article/Supplementary Materials. Further inquiries can be directed to the corresponding author.

## Supplementary Materials

The following supporting information can be downloaded at: https://www.mdpi.com/article/10.3390/foods14203534/s1, Table S1: Calibration curves; Figure S1: Calibration curves; Table S2: TXRF Limits of detection in the analysis of dietary supplements; Table S3: Heavy metals in dietary supplement samples; Table S4: EDIs, THQ, HI, CR, and CCR calculated values for *n* = 45 dietary supplements; Table S5: A PCC analysis; Table S6: Principal Component Analysis; Table S7: Supplements ordered according to their value of Principal Component 1.
