# Supplementary material for: Assessment of Heavy Metals in Mexican Dietary Supplements Using Total X-Ray Fluorescence Spectrometry and Health Risk Evaluation
Source: Foods. 2025 Oct 17;14(20):3534. doi: 10.3390/foods14203534 (PMC12564666; doi:10.3390/foods14203534)
Supplement: Supplementary file 1 [file foods-14-03534-s001.zip › foods-3887577-supplementary.pdf]

Supplementary data 1

Table S1. Calibration curves.

Estándar concentration (ppb)	Lead	Chromium	Arsenic
0	0.02965941	0.01594533	0.06435962
2	0.1865298	0.07507115	0.35125039
20	1.59759482	0.64154226	2.54896502
40	3.62123198	1.3316215	4.79117699
60	5.57446809	1.93051487	6.87805043
80	7.71963151	2.66369259	9.34273332
100	9.75212788	3.40164022	12.1350821

Figure S1. Calibration curves

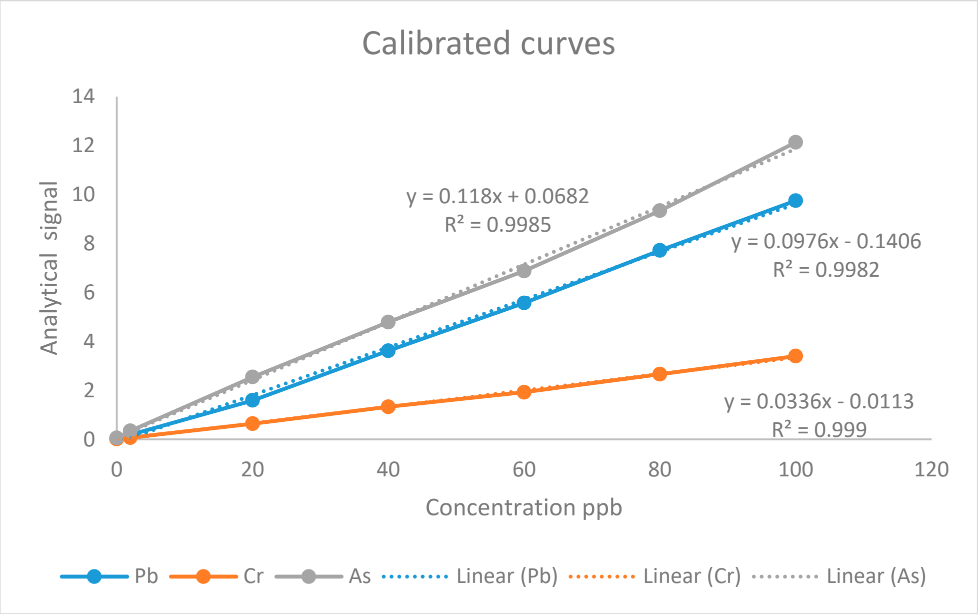

Table S2. TXRF Limits of detection in the analysis of dietary supplements.

Blanks	Lead	Arsenic	Chromium
1	0.074	0.036	0.077
2	0.100	0.08	0.0544
3	0.046	0.033	0.062
4	0.066	0.072	0.080
5	0.057	0.033	0.077
6	0.057	0.022	0.082
7	0.097	0.069	0.079
8	0.062	0.041	0.070
9	0.083	0.041	0.069
10	0.066	0.069	0.056
11	0.043	0.114	0.063
12	0.066	0.052	0.059
13	0.057	0.052	0.063
Standard deviation	0.01744642	0.0252	0.00960997
m	0.0976	0.118	0.0336
**LOD**	**0.54**	**0.64**	**0.86**
**LOQ**	**1.79**	**2.14**	**2.86**

Table S3. Heavy metals in dietary supplement samples.

Sample ID	Pb (mg/Kg)	Cr (mg/Kg)	As (mg/Kg)
M1	4.51 ± 0.66	131.46 ± 0.39	0.21 ± 0.02
V2	1.63 ± 0.02	12.97 ± 0.06	0.31 ± 0.04
M3	1.08 ± 0.17	15.97 ± 0.46	0.46 ± 0.06
V4	1.52 ± 0.07	11.19 ± 0.32	0.26 ± 0.02
M5	1.64 ± 0.14	2.15 ± 0.01	1.34 ± 038
C6	1.75 ± 0.06	8.47 ± 0.42	0.06 ± 0.09
V7	0.82 ± 0.04	2.67 ± 0.06	0.12 ± 0.05
V9	4.20 ± 0.16	30.14 ± 0.17	0.42
V10	2.16 ± 0.06	0.1588 ± 0.0005	< LOD
A11	9.03 ± 0.22	34.68 ± 0.44	11.41 ± 0.65
A12	1.58 ± 0.03	0.3476 ± 0.0001	0.424 ± 0.039
V13	2.55 ± 0.06	6.31 ± 0.15	39.93 ± 0.32
V14	2.51 ± 0.11	0.272 ± 0.001	1.49 ± 0.05
V15	2.25 ± 0.09	< LOD	0.66 ± 0.04
V16	2.98 ± 0.03	135.41 ± 0.10	0.07 ± 0.03
V17	1.98 ± 0.05	8.00 ± 0.33	5.91 ± 0.96
V18	8.17 ± 0.05	29.81 ± 0.34	1.47 ± 0.03
V19	2.59 ± 0.02	150.01 ± 0.03	< LOD
V20	2.64 ± 1.05	65.96 ± 0.33	0.12 ± 0.09
A21	0.59 ± 0.07	1.15 ± 0.08	< LOD
V22	0.39 ± 0.04	0.32083 ± 0.00008	< LOD
S24	0.40 ± 0.02	2.49 ± 0.03	< LOD
V25	0.94 ± 0.07	0.16 ± 0.02	< LOD
V26	0.73 ± 0.4	22.52 ± 0.28	1.08 ± 0.02
S27	0.29 ± 0.02	2.00 ± 0.04	0.23 ± 0.01
S28	1.485 ± 0.002	0.420 ± 0.004	< LOD
S29	1.749 ± 0.009	0.687 ± 0.013	< LOD
S30	1.7857 ± 0.0005	0.632 ± 0.006	< LOD
S31	1.812 ± 0.001	0.466 ± 0.002	< LOD
V32	0.54 ± 0.10	233.20 ± 1.45	0.24 ± 0.06
V33	0.49 ± 0.17	< LOD	0.05 ± 0.02
V34	0.47 ± 0.1	25.84 ± 0.71	0.93 ± 0.05
C35	0.24 ± 0.14	14.52 ± 0.56	0.18 ± 0.02
A36	0.41 ± 0.11	39.56 ± 0.45	0.25 ± 0.05
V37	0.58 ± 0.12	< LOD	< LOD
V38	0.53 ± 0.03	3.09 ± 0.18	0.28 ± 0.11
V39	< LOD	15.15 ± 0.23	< LOD
C40	0.07 ± 0.01	0.08 ± 0.02	1.55 ± 0.25
V41	< LOD	6.95 ± 0.09	37.11
A42	0.39 ± 0.09	< LOD	< LOD

V43	1.39 ± 0.20	24.16 ± 0.32	0.026 ± 0.008
V44	0.86 ± 0.07	127.97 ± 0.48	0.31 ± 0.1
A45	7.20 ±0.61	15.36 ± 0.15	0.47 ± 0.17
A46	4.11 ± 0.17	18.21 ± 0.33	< LOD
A47	6.32 ± 0.17	8.71 ± 0.30	< LOD
**Minimum**	0.0700 ± 0.0005	0.07800 ± 0.00008	0.0260 ± 0.0004
**Maximum**	9.03 ± 1.05	233.20 ± 1.45	39.93 ± 0.96
**Mean**	1.99 ± 0.13	26.88 ± 0.23	2.39 ± 0.11

Results are expressed as the mean value ± *s* (n = 3); *s*, standard deviation; < LOD, values lower than the TXRF limit of detection.

Table S4. EDIs, THQ, HI, CR, and CCR calculated values for n=45 dietary supplements.

ID	ORIGIN	CONC_Pb	CONC_Cr	CONC_As	EDI_Pb	EDI_Cr	EDI_As	THQ_Pb	THQ_Cr	THQ_As	HI	CR_Pb	CR_Cr	CR_As	CCR
A_11	ANIMAL	9.03100000	34.68000000	11.41000000	0.00030963	0.00118903	0.00039120	1.22534116	0.00282326	4.64438356	5.87254799	0.00000263	0.00059451	0.00058680	0.00118395
A_12	ANIMAL	1.58000000	0.34760000	0.42400000	0.00067714	0.00014897	0.00018171	2.67971298	0.00035372	2.15733855	4.83740526	0.00000576	0.00007449	0.00027257	0.00035281
A_21	ANIMAL	0.59000000	1.15000000	0.00000000	0.00025286	0.00049286	0.00000000	1.00065232	0.00117025	0.00000000	1.00182257	0.00000215	0.00024643	0.00000000	0.00024858
A_36	ANIMAL	0.41000000	39.56000000	0.25000000	0.00001406	0.00135634	0.00000857	0.05562948	0.00322054	0.10176125	0.16061128	0.00000012	0.00067817	0.00001286	0.00069115
A_42	ANIMAL	0.39000000	0.00000000	0.00000000	0.00016714	0.00000000	0.00000000	0.66144814	0.00000000	0.00000000	0.66144814	0.00000142	0.00000000	0.00000000	0.00000142
A_45	ANIMAL	7.19600000	15.36000000	0.46680000	0.00092520	0.00197486	0.00006002	3.66136986	0.00468916	0.71253229	4.37859131	0.00000786	0.00098743	0.00009003	0.00108532
A_46	ANIMAL	4.10730000	18.21000000	0.00000000	0.00052808	0.00234129	0.00000000	2.08981996	0.00555922	0.00000000	2.09537918	0.00000449	0.00117064	0.00000000	0.00117513
A_47	ANIMAL	6.32000000	8.71000000	0.00000000	0.00081257	0.00111986	0.00000000	3.21565558	0.00265902	0.00000000	3.21831460	0.00000691	0.00055993	0.00000000	0.00056684
C_35	COMBINED	0.24000000	14.52000000	0.18000000	0.00000343	0.00020743	0.00000257	0.01356817	0.00049252	0.03052838	0.04458907	0.00000003	0.00010371	0.00000386	0.00010760
C_40	COMBINED	0.07000000	0.07800000	1.55000000	0.00000050	0.00000056	0.00001107	0.00197869	0.00000132	0.13144162	0.13342163	0.00000000	0.00000028	0.00001661	0.00001689
C_6	COMBINED	1.75000000	8.47000000	0.05600000	0.00004130	0.00019989	0.00000132	0.16343988	0.00047463	0.01569023	0.17960474	0.00000035	0.00009995	0.00000198	0.00010228
M_1	MINERAL	4.51000000	131.46000000	0.21000000	0.00012886	0.00375600	0.00000600	0.50993694	0.00891836	0.07123288	0.59008818	0.00000110	0.00187800	0.00000900	0.00188810
M_3	MINERAL	1.08000000	15.97000000	0.46000000	0.00002314	0.00034221	0.00000986	0.09158513	0.00081256	0.11702544	0.20942313	0.00000020	0.00017111	0.00001479	0.00018609
M_5	MINERAL	1.64000000	2.15000000	1.34000000	0.00002343	0.00003071	0.00001914	0.09271581	0.00007293	0.22726680	0.32005553	0.00000020	0.00001536	0.00002871	0.00004427
S_24	SYNTHETIC	0.40200000	2.49000000	0.00000000	0.00022971	0.00142286	0.00000000	0.90906719	0.00337847	0.00000000	0.91244566	0.00000195	0.00071143	0.00000000	0.00071338
S_27	SYNTHETIC	0.28700000	2.00000000	0.23000000	0.00000410	0.00002857	0.00000329	0.01622527	0.00006784	0.03900848	0.05530159	0.00000003	0.00001429	0.00000493	0.00001925
S_28	SYNTHETIC	1.48000000	0.42000000	0.00000000	0.00001321	0.00000375	0.00000000	0.05229398	0.00000890	0.00000000	0.05230288	0.00000011	0.00000188	0.00000000	0.00000199
S_29	SYNTHETIC	1.75000000	0.68700000	0.00000000	0.00012500	0.00004907	0.00000000	0.49467275	0.00011652	0.00000000	0.49478927	0.00000106	0.00002454	0.00000000	0.00002560
S_30	SYNTHETIC	1.78000000	0.63200000	0.00000000	0.00012714	0.00004514	0.00000000	0.50315286	0.00010719	0.00000000	0.50326005	0.00000108	0.00002257	0.00000000	0.00002365
S_31	SYNTHETIC	1.81000000	0.46600000	0.00000000	0.00012929	0.00003329	0.00000000	0.51163296	0.00007903	0.00000000	0.51171200	0.00000110	0.00001664	0.00000000	0.00001774
V_10	VEGETABLE	2.16000000	0.15880000	0.00000000	0.00004629	0.00000340	0.00000000	0.18317025	0.00000808	0.00000000	0.18317833	0.00000039	0.00000170	0.00000000	0.00000209
V_13	VEGETABLE	2.55000000	6.31000000	39.93000000	0.00010929	0.00027043	0.00171129	0.43248532	0.00064211	20.31663405	20.74976149	0.00000093	0.00013521	0.00256693	0.00270307
V_14	VEGETABLE	2.51000000	0.27200000	1.49000000	0.00010757	0.00001166	0.00006386	0.42570124	0.00002768	0.75812133	1.18385025	0.00000091	0.00000583	0.00009579	0.00010253
V_15	VEGETABLE	2.25000000	0.00000000	0.66000000	0.00106071	0.00000000	0.00031114	4.19765166	0.00000000	3.69393346	7.89158513	0.00000902	0.00000000	0.00046671	0.00047573
V_16	VEGETABLE	2.98000000	135.41000000	0.07000000	0.00008514	0.00386886	0.00000200	0.33694281	0.00918633	0.02374429	0.36987343	0.00000072	0.00193443	0.00000300	0.00193815
V_17	VEGETABLE	1.98000000	8.00000000	5.91000000	0.00002829	0.00011429	0.00008443	0.11193738	0.00027136	1.00234834	1.11455708	0.00000024	0.00005714	0.00012664	0.00018403
V_18	VEGETABLE	8.17000000	29.81000000	1.47000000	0.00011671	0.00042586	0.00002100	0.46188302	0.00101117	0.24931507	0.71220925	0.00000099	0.00021293	0.00003150	0.00024542
V_19	VEGETABLE	2.60000000	150.01000000	0.00000000	0.00022286	0.01285800	0.00000000	0.88193085	0.03053041	0.00000000	0.91246127	0.00000189	0.00642900	0.00000000	0.00643089
V_2	VEGETABLE	1.63000000	12.97000000	0.31000000	0.00002794	0.00022234	0.00000531	0.11058056	0.00052794	0.06309198	0.17420047	0.00000024	0.00011117	0.00000797	0.00011938
V_20	VEGETABLE	2.64000000	65.96000000	0.12000000	0.00013577	0.00339223	0.00000617	0.53729941	0.00805461	0.07326810	0.61862212	0.00000115	0.00169611	0.00000926	0.00170653
V_22	VEGETABLE	0.39000000	0.32083000	0.00000000	0.00013929	0.00011458	0.00000000	0.55120678	0.00027207	0.00000000	0.55147885	0.00000118	0.00005729	0.00000000	0.00005848
V_25	VEGETABLE	0.94000000	0.16000000	0.00000000	0.00002014	0.00000343	0.00000000	0.07971298	0.00000814	0.00000000	0.07972112	0.00000017	0.00000171	0.00000000	0.00000189
V_26	VEGETABLE	0.73000000	22.52000000	1.08000000	0.00001043	0.00032171	0.00001543	0.04126984	0.00076389	0.18317025	0.22520398	0.00000009	0.00016086	0.00002314	0.00018409
V_32	VEGETABLE	0.54000000	233.20000000	0.24000000	0.00004629	0.01998857	0.00002057	0.18317025	0.04746145	0.24422701	0.47485871	0.00000039	0.00999429	0.00003086	0.01002554
V_33	VEGETABLE	0.49000000	0.00000000	0.05000000	0.00001050	0.00000000	0.00000107	0.04155251	0.00000000	0.01272016	0.05427267	0.00000009	0.00000000	0.00000161	0.00000170
V_34	VEGETABLE	0.47000000	25.84000000	0.93000000	0.00000671	0.00036914	0.00001329	0.02657099	0.00087650	0.15772994	0.18517744	0.00000006	0.00018457	0.00001993	0.00020456
V_37	VEGETABLE	0.58000000	0.00000000	0.00000000	0.00002486	0.00000000	0.00000000	0.09836921	0.00000000	0.00000000	0.09836921	0.00000021	0.00000000	0.00000000	0.00000021
V_38	VEGETABLE	0.53000000	3.09000000	0.28000000	0.00002271	0.00013243	0.00001200	0.08988911	0.00031444	0.14246575	0.23266930	0.00000019	0.00006621	0.00001800	0.00008441
V_39	VEGETABLE	0.00000000	15.15000000	0.00000000	0.00000000	0.00077914	0.00000000	0.00000000	0.00185002	0.00000000	0.00185002	0.00000000	0.00038957	0.00000000	0.00038957
V_4	VEGETABLE	1.52000000	11.19000000	0.26000000	0.00003257	0.00023979	0.00000557	0.12889759	0.00056935	0.06614481	0.19561175	0.00000028	0.00011989	0.00000836	0.00012853
V_41	VEGETABLE	0.00000000	6.95000000	37.11000000	0.00000000	0.00029786	0.00159043	0.00000000	0.00070724	18.88180039	18.88250763	0.00000000	0.00014893	0.00238564	0.00253457
V_43	VEGETABLE	1.39000000	24.16000000	0.02600000	0.00006553	0.00113897	0.00000123	0.25932159	0.00270441	0.01455186	0.27657786	0.00000056	0.00056949	0.00000184	0.00057188
V_44	VEGETABLE	0.86000000	127.97000000	0.31000000	0.00001474	0.00219377	0.00000531	0.05834312	0.00520895	0.06309198	0.12664405	0.00000013	0.00109689	0.00000797	0.00110498
V_7	VEGETABLE	0.82000000	2.67000000	0.11800000	0.00004217	0.00013731	0.00000607	0.16688845	0.00032604	0.07204697	0.23926146	0.00000036	0.00006866	0.00000910	0.00007812
V_9	VEGETABLE	4.20000000	30.14000000	0.42000000	0.00019800	0.00142089	0.00001980	0.78356164	0.00337379	0.23506849	1.02200393	0.00000168	0.00071044	0.00002970	0.00074183

Table S5: A PCC analysis.

Pearson correlation coefficients n = 45			
Prob > |r| assuming H0: Rho=0			
	**EDI_Cr**	**EDI_As**	**HI**
**EDI_Pb** P_value	**0.01466**	**0.02934**	**0.26055**
	0.9238	0.8483	0.0839
**EDI_Cr** P_value		**-0.0808**	**-0.07269**
		0.5978	0.6351
**EDI_As** P_value			**0.97269**
			<.0001

Table S6: Principal Component Analysis
Total variance explained

CP	Autovalor	DIFFERENCE	PROPORTION	CUMULATIVE
**1**	2.02507358	1.0008684	0.5063	0.5063
**2**	1.02420518	0.07348394	0.2561	**0.7623**
**3**	0.95072124	0.95072124	0.2377	1
**4**	0		0	1

Table S7. Supplements ordered according to their value of Principal Component 1.

ID	Prin1	Prin2	EDI_Pb	EDI_Cr	EDI_As	HI
V_32	-1.00255	3.542930000	0.000046286	0.019989000	0.000020571	0.47490
V_19	-0.62860	2.564430000	0.000222857	0.012858000	0.000000000	0.91250
V_39	-0.60805	-0.534900000	0.000000000	0.000779000	0.000000000	0.00190
V_44	-0.60599	-0.204490000	0.000014743	0.002194000	0.000005314	0.12660
C_35	-0.57697	-0.643910000	0.000003429	0.000207000	0.000002571	0.04460
A_36	-0.57064	-0.379700000	0.000014057	0.001356000	0.000008571	0.16060
S_27	-0.56817	-0.679090000	0.000004100	0.000029000	0.000003286	0.05530
S_28	-0.56724	-0.658320000	0.000013214	0.000004000	0.000000000	0.05230
V_33	-0.56684	-0.666820000	0.000010500	0.000000000	0.000001071	0.05430
V_16	-0.56511	0.334600000	0.000085143	0.003869000	0.000002000	0.36990
V_25	-0.55728	-0.639370000	0.000020143	0.000003000	0.000000000	0.07970
V_37	-0.55042	-0.627140000	0.000024857	0.000000000	0.000000000	0.09840
C_40	-0.54211	-0.696790000	0.000000500	0.000001000	0.000011071	0.13340
V_34	-0.53493	-0.604410000	0.000006714	0.000369000	0.000013286	0.18520
V_2	-0.53148	-0.574270000	0.000027943	0.000222000	0.000005314	0.17420
C_6	-0.52729	-0.541190000	0.000041300	0.000200000	0.000001322	0.17960
V_4	-0.52433	-0.558050000	0.000032571	0.000240000	0.000005571	0.19560
M_3	-0.52399	-0.563960000	0.000023143	0.000342000	0.000009857	0.20940
V_26	-0.51988	-0.604540000	0.000010429	0.000322000	0.000015429	0.22520
V_10	-0.51976	-0.567620000	0.000046286	0.000003000	0.000000000	0.18320
V_43	-0.51936	-0.281260000	0.000065529	0.001139000	0.000001226	0.27660
V_38	-0.51032	-0.608900000	0.000022714	0.000132000	0.000012000	0.23270
V_7	-0.50572	-0.552930000	0.000042171	0.000137000	0.000006069	0.23930
M_1	-0.48355	0.430280000	0.000128857	0.003756000	0.000006000	0.59010
M_5	-0.47850	-0.629770000	0.000023429	0.000031000	0.000019143	0.32010
V_20	-0.46271	0.374300000	0.000135771	0.003392000	0.000006171	0.61860
S_29	-0.40808	-0.342170000	0.000125000	0.000049000	0.000000000	0.49480
S_30	-0.40489	-0.337100000	0.000127143	0.000045000	0.000000000	0.50330
S_31	-0.40148	-0.333660000	0.000129286	0.000033000	0.000000000	0.51170
V_22	-0.38942	-0.289470000	0.000139286	0.000115000	0.000000000	0.55150
V_18	-0.34849	-0.292840000	0.000116714	0.000426000	0.000021000	0.71220
A_42	-0.34621	-0.236610000	0.000167143	0.000000000	0.000000000	0.66140
S_24	-0.29650	0.228170000	0.000229714	0.001423000	0.000000000	0.91240
V_9	-0.26456	0.135500000	0.000198000	0.001421000	0.000019800	1.02200
A_21	-0.23708	0.100150000	0.000252857	0.000493000	0.000000000	1.00180
V_17	-0.21867	-0.616420000	0.000028286	0.000114000	0.000084429	1.11460
V_14	-0.18240	-0.414530000	0.000107571	0.000012000	0.000063857	1.18390
A_46	0.10584	1.236240000	0.000528081	0.002341000	0.000000000	2.09540
A_47	0.54857	1.765510000	0.000812571	0.001120000	0.000000000	3.21830
A_45	0.92074	2.234920000	0.000925200	0.001975000	0.000060017	4.37860
A_12	1.09192	1.146000000	0.000677143	0.000149000	0.000181714	4.83740
A_11	1.35408	0.296350000	0.000309634	0.001189000	0.000391200	5.87250
V_15	2.15260	2.134010000	0.001060714	0.000000000	0.000311143	7.89160
V_41	5.62288	-1.052830000	0.000000000	0.000298000	0.001590429	18.88250
V_13	6.25296	-0.790350000	0.000109286	0.000270000	0.001711286	20.74980
